# Role of postoperative radiotherapy in resected adenoid cystic carcinoma of the head and neck

**DOI:** 10.1186/s13014-022-02165-5

**Published:** 2022-12-01

**Authors:** Seo Hee Choi, Andrew Jihoon Yang, Sun Och Yoon, Hye Ryun Kim, Min Hee Hong, Se-Heon Kim, Eun Chang Choi, Ki Chang Keum, Chang Geol Lee

**Affiliations:** 1 Department of Radiation Oncology, Yongin Severance Hospital, Yonsei University College of Medicine, Yongin, Gyeonggi-Do Korea; 2 Department of Radiation Oncology, Yonsei Cancer Center, Heavy Ion Therapy Research Institute, Yonsei University College of Medicine, Seoul, Korea; 3 Department of Inpatient Medicine, Yongin Severance Hospital, Yonsei University College of Medicine, Yongin, Gyeonggi-Do Korea; 4 Department of Pathology, Yonsei University College of Medicine, Severance Hospital, Seoul, Korea; 5 Division of Medical Oncology, Department of Internal Medicine, Yonsei Cancer Center, Yonsei University College of Medicine, Seoul, Korea; 6 Department of Otorhinolaryngology, Yonsei Cancer Center, Yonsei University College of Medicine, Seoul, Korea

**Keywords:** Adenoid cystic carcinoma, Postoperative radiotherapy, Radiation, Prognosis, Local recurrence

## Abstract

**Purpose:**

Although postoperative radiotherapy (PORT) is demonstrably effective in local control of head and neck adenoid cystic carcinoma (HNACC), its application is controversial and the subset of patients who would benefit most from PORT is unknown. Herein, we analyzed the data of HNACC patients to clarify the role of PORT.

**Methods:**

We retrospectively reviewed 187 patients with nonmetastatic HNACC who underwent surgical resection between 2005 and 2019. The study endpoints were locoregional failure-free survival (LRFFS), progression-free survival (PFS), and overall survival (OS). Subgroup analysis and recursive partitioning analysis (RPA) were performed to identify patients most likely to benefit from PORT.

**Results:**

With a median follow-up of 84.7 months, the 5-year LRFFS, PFS, and OS were 70.0%, 52.6%, and 86.4%, respectively. Survival was significantly shorter in patients who experienced local failure than in those who did not (5-year OS: 88.1% vs. 80.5%, *P* = 0.001). The local failure rate was significantly lower in patients who underwent PORT (16.9% vs. 31.0%, *P* = 0.021), despite the high frequency of adverse factors. Especially, significant decreases in local failure and similar OS rates could be obtained after PORT among patients with positive margins, T2-4 stage disease, and minor salivary gland tumors. The RPA model for PFS categorized patients into four groups according to three prognostic factors (T-stage, location, and sex). The RPA model for LRFFS and OS suggested three groups based on two factors (T-stage, margin for LRFFS; T-stage, grade 3 for OS).

**Conclusion:**

PORT could prevent dismal survival, while significantly reducing local failures in high-risk HNACC patients.

**Supplementary Information:**

The online version contains supplementary material available at 10.1186/s13014-022-02165-5.

## Introduction

Adenoid cystic carcinoma (ACC) is a rare malignancy of the salivary glands of the head and neck, accounting for approximately 1% of head and neck malignancies and 10% of malignant tumors of the salivary glands [1]. It is the second most common malignant salivary gland tumor in the parotid gland and the most common malignant subtype in the submandibular and sublingual glands [2, 3]. Histologically, the three principal growth patterns of ACC are solid, cribriform, and tubular; the solid pattern, which is known to be more aggressive, is characterized by frequent local relapse and distant failure. ACCs are characterized by a poor prognosis due to slow but locally aggressive growth and a high rate of distant metastases even years after the initial diagnosis.

Surgical resection followed by radiation is the standard of care for the management of the majority of ACC cases, irrespective of anatomic location or gland subtype. However, the preponderance of perineural invasion (PNI) and advanced T classification at presentation often limit the scope of surgery and are responsible for the high local recurrence rate even after surgery. Consequently, postoperative radiotherapy (PORT) can be considered as an important adjuvant strategy for the treatments of ACCs. PORT has been shown to increase locoregional control at all stages of the disease [4, 5], and a survival benefit can be anticipated even in the early stage [6]. Based on recent studies, the American Society of Clinical Oncology (ASCO) and National Comprehensive Cancer Network (NCCN) guidelines recommend PORT for all patients with resected ACC [7, 8]. However, various conflicting results [1, 9–11] have led to ambiguity regarding the improvement in local control and survival imparted by PORT in patients with ACC. Further research is also needed to determine the subset of patients who would derive the greatest benefit from PORT. Moreover, although the probability of lymph node metastasis is low, it is controversial whether elective neck treatment is necessary in cN0 ACCs.

However, the role of PORT must continue to be specifically explored, in situations where effective adjuvant therapies, including systemic chemotherapy, are limited. We postulated that PORT can improve survival duration as well as control of locoregional recurrence in a certain subset of patients with ACC. In this study, we analyzed patients with head and neck ACCs who had received surgical resection with or without PORT to clarify the effects of the former on the prognosis. We attempted to provide individualized suggestions for PORT in patients with ACCs by reviewing our treatment experience over a long period.

## Materials and methods

### Patients

A total of 228 patients were pathologically diagnosed with nonmetastatic head and neck ACC (major or minor glands) and treated at Yonsei Cancer Center between January 2005 through December 2019. We only included patients who had undergone surgery with the intent of curative resection (± PORT) in the analysis. The following patients were excluded: 11 patients who underwent initial surgery at other hospitals without an accurate record of the baseline data, 14 with initial distant metastases, 10 patients were lost to follow-up within 3 months of treatment, and 6 patients who did not undergo surgery (and received definitive radiotherapy instead). Overall, 187 consecutive patients who had undergone surgical resection for head and neck ACC were included in this study. The medical records of these patients were collected and analyzed retrospectively. The study was approved by our institutional review board (No. 9–2022-0100), and requirement for informed consent was waived.

### Treatment policy

Surgical resection was considered first in resectable cases. Surgery involved resection of the primary tumor with appropriate reconstruction. A selective approach was employed for neck dissection. The extent of lymph node dissection was determined at the surgeon’s discretion, mainly based on the patient’s medical history, clinical palpation, imaging data, and intraoperative exploration. Generally, the indications for PORT included stage III/IV cancer and adverse pathological factors such as close/positive margins, PNI, high histologic grade, and R2 resected tumors. All tumors were staged according to using the American Joint Committee on Cancer (AJCC) 8th edition. Histologic grade was classified into the following three grades [12, 13]: Grade I, tumors with tubular and cribriform areas but without solid components; Grade II, cribriform tumors that were either pure or mixed with less than 30% of solid areas; and Grade III, tumors with a predominantly solid pattern. The median prescription dose delivered to the post-operative bed was 63 Gy, which was increased to 66 Gy if the margins were positive. The high-risk clinical target volume (CTV) for the PORT field included the pre-operative gross disease and the post-operative tumor bed at the primary site, in addition to any nodal regions with disease involvement. In some node-negative patients, the adjacent lymph node areas were also included in the low-risk CTV, at the radiation oncologist’s discretion. In patients with PNI, the field of radiation encompassed the path of the nerves to the skull base to cover potential spread to the nerves. The prescribed dose delivered to the intermediate- and low-risk CTV was 45–60 Gy. The planning target volume was obtained by adding a margin of 3 to 5 mm to the CTV. Patients were treated with 3-dimensional conformal radiotherapy (3D-CRT) or intensity-modulated radiotherapy (IMRT), depending on the period of treatment. Neck treatment denoted neck lymph node dissection or neck irradiation, which was performed therapeutically in all node-positive cases or prophylactically in some node-negative cases.

### Follow-up for treatment outcomes and statistical analysis

After finishing the first session of treatment, patients were followed up every 3 months for the first 2 years, and every 6 months thereafter, until death. Pathologic confirmation or radiographic evidence (computed tomography, magnetic resonance imaging, or positron emission tomography/computed tomography) was necessary to determine the presence and location of recurrence. The pattern of the first recurrence was classified as local, regional, locoregional, and distant failure. Locoregional failure-free survival (LRFFS; i.e., the time from surgery to the first appearance of locoregional failure), local failure-free survival (LFFS; i.e., the time from surgery to the first appearance of local failure), progression-free survival (PFS; i.e., the time from surgery to any type of failure), and overall survival (OS; i.e., the time from surgery to death due to any cause or last follow-up) were calculated using Kaplan–Meier estimates. Univariate and multivariate Cox regression analyses was performed to confirm the role of clinicopathological factors and PORT at each survival endpoint (LRFFS, PFS, and OS). We included age, sex, primary tumor location, histological grade, tumor size, T stage, N stage, extracapsular extension, surgical margin, PNI, lymphovascular invasion (LVI), and PORT in the Cox model. Only those factors that were significant in the univariate analysis and had no multicollinearity were included in the multivariate model.

We performed the following analyses to identify patients who were most likely to benefit from PORT. First, recursive partitioning analysis (RPA) was performed based on the same variables, except PORT. Recursive partitioning is a method of building decision trees to model predictors [14], which uses Kaplan–Meier estimates of survival and modified Wilcoxon tests to establish branches in the decision tree [15, 16]. It examines all possible cut-offs for all variables entered into the model. These cut-off values are used to divide the dataset into two relatively homogeneous populations that differ significantly with respect to survival. Final RPA models were established for LRFFS, PFS, and OS, respectively. Second, forest plots were generated to summarize the adjusted hazard ratios (HRs) with 95% confidence intervals (CIs) for the validated predictors of LRFFS, PFS, and OS only in the PORT group.

Statistical analyses were conducted using SPSS Statistics, version 25 (IBM Corp) or the party package [17] in R version 4.2.1 (R Foundation for Statistical Computing). All P values were two-sided with statistical significance set at *P* < 0.05.

## Results

### Patients and survival outcomes

Overall, 82 patients with ACC of the major salivary glands (parotid gland, 15.5%; submandibular gland, 19.8%; and sublingual gland, 8.6%) and 105 patients with ACC of the minor salivary glands were included in this study. Sixty-one patients underwent surgical resection alone, 124 patients received PORT (± concurrent chemotherapy), and 2 patients underwent preoperative RT. A median dose of 63.0 Gy was prescribed to patients with RT, and elective lymph node irradiation was performed in 59.5% of patients. Overall neck treatment was performed in 104 patients: 90 at N0 stage (elective aim), 6 at N1 stage (therapeutic aim), and 8 at N2 stage (therapeutic aim). The proportion of patients according to the type of neck treatment is shown in Table 1. All other patient characteristics are also detailed in Table 1.

**Table 1:** Patients’ characteristics

No	%	
Sex		
Male	81	43.3
Female	106	56.7
Location		
Parotid	29	15.5
Submandibular	37	19.8
Sublingual	16	8.6
Minor salivary gland	105	56.1
Histologic grade		
1	76	40.6
2	77	41.2
3	20	10.7
Unknown	14	7.5
Tumor size (cm)		
Median 2.5 (0.4–9.0)		
T stage		
T1	52	0.5
T2	62	27.8
T3	32	33.2
T4	40	17.1
Tx	1	21.4
N stage		
N0	173	92.5
N1	6	3.2
N2	8	4.3
Extracapsular extension		
No	176	94.1
Yes	11	5.9
Lymph node dissection		
Not performed	131	70.1
Performed	56	29.9
Surgical margin		
Positive	104	55.6
Close	57	30.5
Negative	26	13.9
PNI		
No	95	50.8
Yes	92	49.2
LVI		
No	156	83.4
Yes	31	16.6
Nerve invasion		
No	173	92.5
Yes	14	7.5
Bone invasion		
No	146	78.0
Yes	41	21.9
Treatment type		
Surgery only	61	32.6
Surgery + postop RT	106	56.7
Surgery + postop CCRT	16	8.6
Preop CTx + surgery + postop RT	2	1.1
Preop CCRT + surgery	1	0.5
Preop RT + surgery	1	0.5
RT technique		
3D CRT	22	17.5
IMRT	100	79.4
Unknown	4	3.2
RT field		
Tumor bed	47	37.3
Tumor bed + elective LN area	75	59.5
Unknown	4	3.2
RT dose (total, Gy)		
Median 63.0 (36.0–70.4)		
Neck treatment		
Not performed	83	44.4
Performed	104	55.6
Aim of neck treatment		
Therapeutic	14	13.5
Elective	90	86.5
Type of neck treatment		
Elective neck dissection	29	27.9
Elective neck irradiation	47	45.2
Elective neck dissection + irradiation	14	13.5
Therapeutic neck irradiation	1	0.9
Therapeutic neck dissection + irradiation	13	12.5

*PNI* perineural invasion, *LVI* lymphovascular invasion, *RT* radiotherapy, *CCRT* concurrent chemoradiotherapy, *CTx* chemotherapy, *3D CRT* 3-dimensional conformal radiotherapy, *IMRT* intensity-modulated radiotherapy, *LN* lymph node

**Fig. 1:**
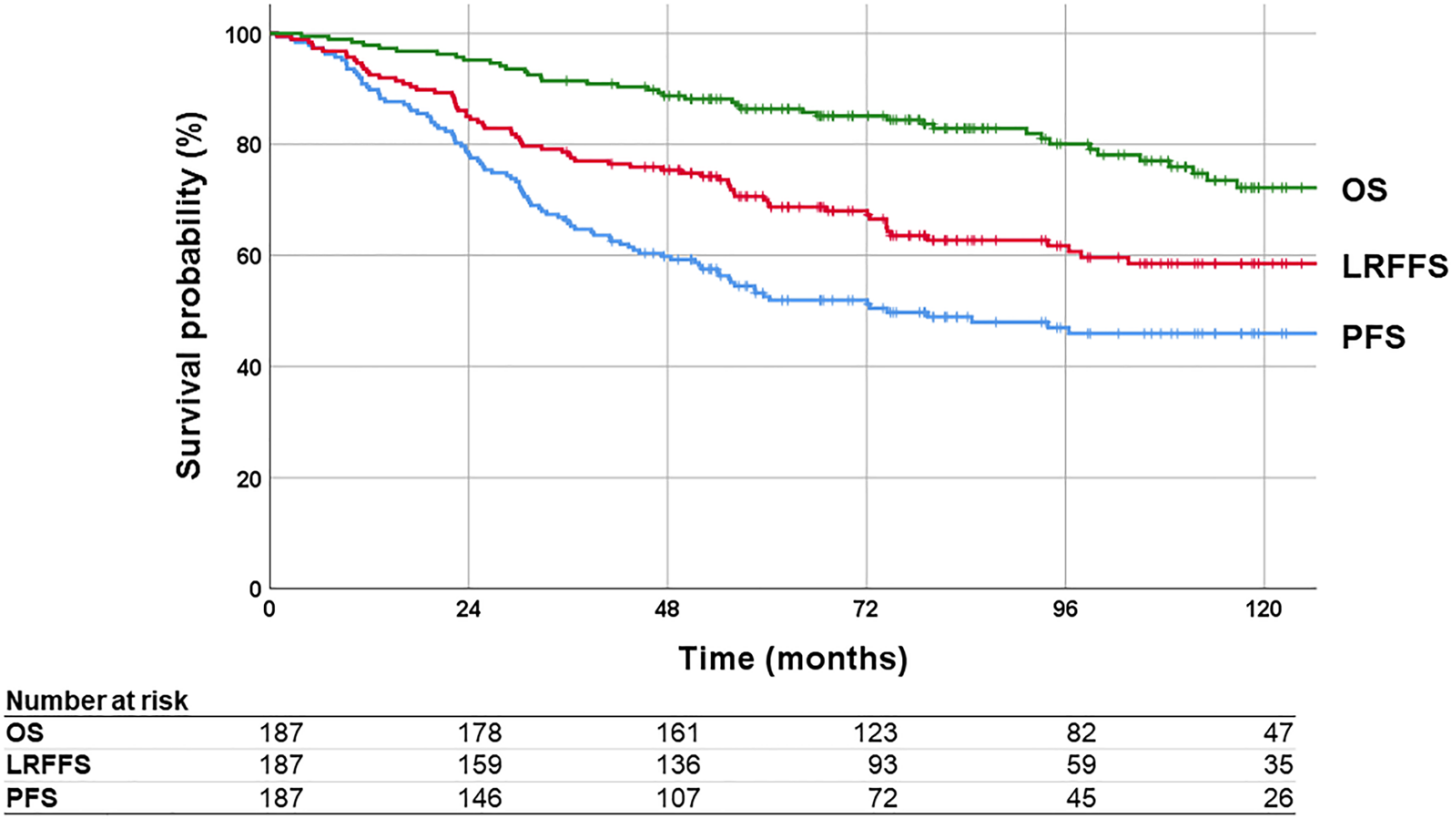
Kaplan–Meier curves of progression-free survival (PFS), locoregional failure-free survival (LRFFS), and overall survival (OS) for all patients

**Fig. 2:**
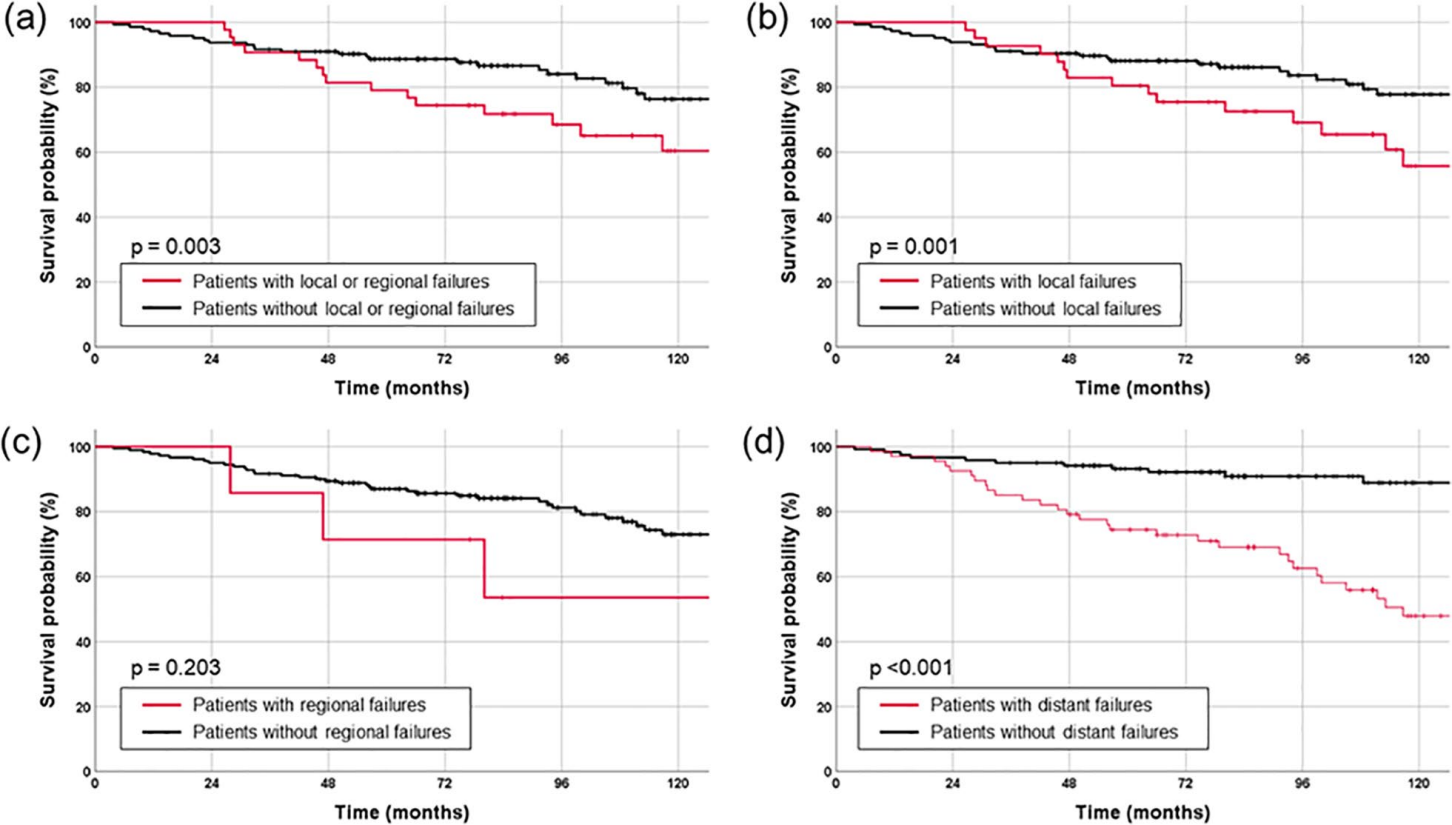
Kaplan–Meier estimates for overall survival depending on whether the patients had experienced **a** locoregional, **b** local, **c** regional, or **d** distant failure

The median follow-up duration was 84.7 months (range: 3.8–204.4) from the date of surgery. The 5-year LRFFS, PFS, and OS were 70.0%, 52.6%, and 86.4%, respectively (Fig. 1). Locoregional failure occurred in 46 cases (24.6%; local failure only, 39; regional failure only, 5; and local + regional failure, 2), distant failure occurred in 67 cases (35.8%), and 47 patients (25.1%) died. The duration of survival of patients with local or distant failure was significantly shorter than that of patients who did not experience local or distant failure (5-year OS: 88.1% vs. 80.5%, *P* = 0.001; 93.2% vs. 74.5%, *P* < 0.001). The occurrence of regional failure had no significant effect on survival (5-year OS 71.4% vs. 87.0%, *P* = 0.203) (Fig. 2). Salvage treatment was performed in 79.5% of patients with recurrences (local treatment (surgery and/or RT), n = 57; systemic chemotherapy alone, n = 13, respectively). The 5-year OS rates of patients with local treatments and no salvage treatment were 80.5% and 86.7%, respectively, which were higher than those of patients with systemic chemotherapy (61.5%) (local vs. chemotherapy, *P* = 0.014; local vs. none, *P* = 0.511; chemotherapy vs. none, *P* = 0.372) (Additional file 1: Fig. S1). The results of univariate and multivariate analyses for the relationship of each survival endpoint with the other prognostic factors are shown in Additional file 1: Tables S1 and S2.

Patients were stratified based on whether or not PORT was performed. The proportion of patients with an advanced T-stage or node-positive tumor, positive resection margin, PNI, LVI, and bone invasion was significantly greater in the PORT group compared to that in the non-PORT group (Table 2). Despite the higher frequency of poor prognostic factors, the incidence of local failure in patients who received PORT was significantly lower than that in patients who did not receive PORT. Contrarily, there was no difference in the regional failure rate, while the distant failure rate was significantly lower in the non-PORT group [Additional file 1: Table S3]. There was no significant difference in the LRFFS (5-year 69.6% vs. 70.7%, *P* = 0.948) or LFFS (5-year, 68.7% vs. 73.2%; *P* = 0.807), but PFS and OS were significantly lower in patients who received PORT (5-year PFS 46.6% vs. 64.2%, *P* = 0.023; 5-year OS 81.1% vs. 96.8%, *P* = 0.002).

**Table 2:** Comparison of basic characteristics between patients who underwent postoperative radiotherapy [PORT (+)] and those who did not undergo postoperative radiotherapy [PORT (−)]

	PORT (+)		PORT (−)		*P* value
	No	%	No	%	
Sex					
Male	57	46.0	24	38.1	0.304
Female	67	54.0	39	61.9	
Location					
Parotid	17	13.7	12	19.0	0.268
Submandibular	24	19.4	13	20.6	
Sublingual	8	6.5	8	12.7	
Minor salivary gland	75	60.5	30	47.6	
Histologic grade					
Grade 1	43	34.7	27	42.9	0.395
Grade 2	54	43.5	22	34.9	
Grade 3	13	10.5	4	6.3	
Unknown	14	11.3	10	15.9	
T stage					
T1	1	0.8	0	0.0	< 0.001
T2	20	16.1	32	50.8	
T3	42	33.9	20	31.7	
T4	27	21.8	5	7.9	
N stage					
N0	110	88.7	63	100.0	0.021
N1	6	4.8	0	0.0	
N2	8	6.5	0	0.0	
Lymph node dissection					
Not performed	90	72.6	41	65.1	0.290
Performed	34	27.4	22	34.9	
Surgical margin					
Positive	86	69.4	18	28.6	< 0.001
Close	29	23.4	28	44.4	
Negative	9	7.3	17	27.0	
Positive margin					
No	38	30.6	45	71.4	< 0.001
Yes	86	69.4	18	28.6	
PNI					
No	52	41.9	43	68.3	0.001
Yes	72	58.1	20	31.7	
LVI					
No	98	79.0	58	92.1	0.024
Yes	26	21.0	5	7.9	
Nerve invasion					
No	113	91.1	60	95.2	0.313
Yes	11	8.9	3	4.8	
Bone invasion					
No	90	72.6	56	88.9	0.011
Yes	34	27.4	7	11.1	

*PORT* postoperative radiotherapy, *PNI* perineural invasion, *LVI* lymphovascular invasion

In patients with positive margin tumors, the local failure rate decreased significantly after PORT (20.9% vs. 61.1%, *P* = 0.001), and there was no difference in the OS (5-year 77.5% vs. 94.4%, *P* = 0.149). Moreover, patients with T2-4 stage tumors experienced a significant decrease in the local failure rate after PORT (5-year 20.2% vs. 45.2%, *P* = 0.005), and there was no significant difference in the OS (5-year 77.5% vs. 96.8%, *P* = 0.085). In patients with minor salivary gland tumors, the local failure rate decreased significantly after PORT (0.0% vs. 16.0%, *P* = 0.008), and there was no significant difference in the OS (5-year, 85.0% vs. 100.0%; *P* = 0.085).

### RPA model for each survival endpoint in the entire cohort

**Fig. 3:**
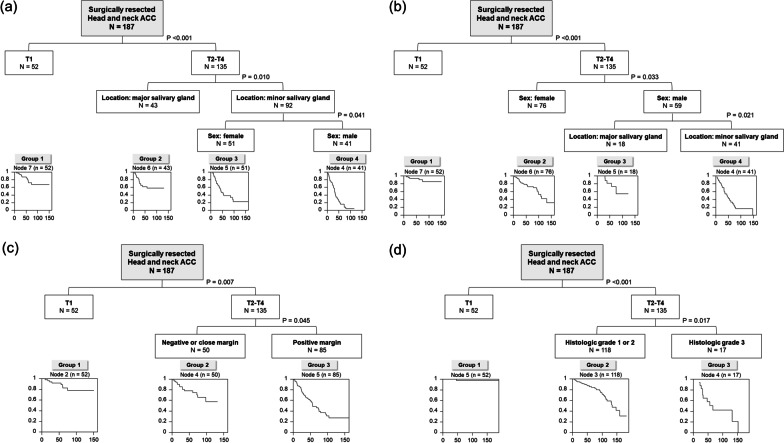
Prognostic grouping of all patients based on recursive partitioning analysis for predicting **A** progression-free survival (PFS), **B** local failure-free survival (LFFS), **C** locoregional failure-free survival (LRFFS), and **D** overall survival (OS). *ACC* adenoid cystic carcinoma

Four RPA models were generated based on each of the endpoints (PFS, LFFS, LRFFS, and OS). The final RPA model for PFS categorized patients into 4 risk groups according to three prognostic factors [T-stage (≥ 2), location (major salivary gland or not), and sex]: group 1, low risk; group 2, intermediate-low risk; group 3, intermediate-high risk; and group 4, high risk (Fig. 3A). The corresponding 5-year PFS rates of groups 1, 2, 3, and 4 were 79.6%, 62.6%, 44.2%, and 17.6%, respectively. The RPA model for LFFS categorized patients into four risk groups using the same prognostic factor with the model for PFS (Fig. 3B). The RPA model for LRFFS categorized patients into 3 risk groups according to two prognostic factors [T-stage (≥ 2) and positive resection margin]: group 1, low risk; group 2, intermediate risk; and group 3, high risk (Fig. 3C). The corresponding 5-year LRFFS rates of groups 1, 2, and 3 were 87.8%, 80.0%, and 53.4%, respectively. The RPA model for OS categorized patients into 3 risk groups according to two prognostic factors [T stage (≥ 2) and histological grade (grade 3 or not)]: group 1, low risk; group 2, intermediate risk; and group 3, high risk (Fig. 3D). The corresponding 5-year OS rates for groups 1, 2, and 3 were 98.0%, 86.6%, and 52.9%, respectively.

### Subgroup analysis of patients who received PORT

**Fig. 4:**
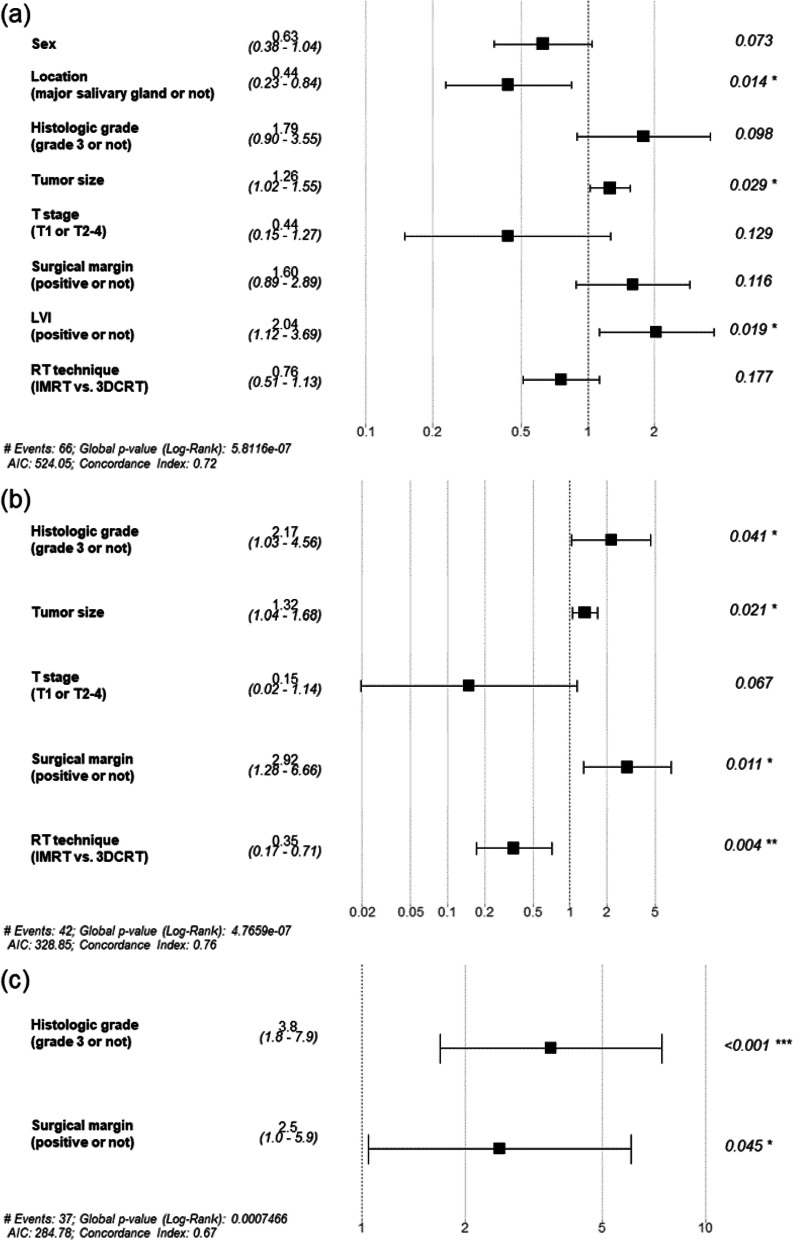
Forest plot analysis of (**A**) progression-free survival (PFS), **B** locoregional failure-free survival (LRFFS) and **C** overall survival (OS) in 124 patients who received postoperative radiotherapy

A total of 124 patients received PORT. The 5-year PFS, LRFFS, and OS of this group were 46.6%, 69.6%, and 81.1%, respectively. Akin to the total study cohort, the survival duration of patients with local or distant failure was significantly shorter than that of those without such failure (5-year OS 71.4% vs. 83.2%, *P* = 0.002; 70.9% vs. 88.6%, *P* < 0.001). The occurrence of regional failure had no significant effect on survival (5-year OS: 60.0% vs. 82.0%, *P* = 0.139). Univariate analysis revealed that major salivary gland location, histological grade < 3, small tumor size, T1 stage, and absence of LVI were significantly associated with the PFS (all *P* < 0.05). Location (major salivary gland), tumor size, and LVI were independently significant factors associated with the PFS, according to the multivariate analysis (*P* = 0.014, 0.029, and 0.019, respectively). Univariate analysis revealed that major salivary gland location, histological grade < 3, tumor size, T1 stage, negative resection margin, and IMRT (vs. 3D-CRT) were significantly associated with a favorable LRFFS (all *P* < 0.05). Histological grade, tumor size, resection margin, and RT technique were significant factors that were independently associated with the LRFFS, as revealed by multivariate analysis (*P* = 0.041, 0.021, 0.011, and 0.004, respectively). Both histological grade and resection margin were significant prognostic factors for LRFFS in the multivariate analyses of OS (*P* = 0.004 and 0.045, respectively), and also served as significant prognostic factors for all three endpoints. The forest plots for each endpoint and the HRs are shown in Fig. 4.

## Discussion

Our study found that regional failure did not affect survival, but the incidence of local failure resulted in significantly shorter survival in patients with ACC. Accordingly, PORT conferred a survival benefit, while significantly reducing local failure. Although we could not demonstrate that PORT prolonged survival by a significant extent, we confirmed the possibility of prognosis improvement in a specific patient subset. Tumors with a grade of T2 or higher, positive margins, and minor salivary gland location should be strongly considered for intensified local therapies (i.e., the addition of PORT, or radiation dose escalation) even after surgical resection. Since the survival can be significantly worse in patients who possess multiple risk factors, including the above-mentioned prognostic indicators, closer monitoring during follow-up is needed. Thus, eventually, surgeons and radiologists can screen patients with ACC who would benefit the most from PORT while providing them with sound advice on adjuvant treatment. We believe that our RPA model for each survival endpoint will also be immensely helpful in determining follow-up plans and treatment strategies.

ACC, which accounts for 3–5% of all head and neck malignancies, is characterized by a slow and persistent growth rate, local invasiveness, low probability of lymphatic spread, and frequent lung metastases. According to previous studies, the 5-year OS, 10-year OS, and 5-year PFS rates were approximately 64–91%, 37–65%, and 50–76%, comparable to those of our study (5-year OS: 86.4% and 5-year PFS: 46.9%). After radical surgery, the local control rate is known to range from 30–70%. Local recurrence is known as one of the principal patterns of treatment failure in patients with ACC. Similarly, a local recurrence rate of 21.9% was observed in our study. Consequently, the key to improving the efficacy of treatment in patients with ACC is to reduce local recurrence. Our finding that the survival of patients who experienced local failure was significantly poor supports this hypothesis.

In an effort to better characterize ACC, studies have attempted to identify the relationships between clinicopathological predictors including stage, clinical presentation, treatment modality and local control, distant metastases, and survival. In our study, we found that T stage, location, sex, margin, histologic grade, and LVI are significant prognostic factors, both in the entire study cohort and in the subgroup of patients who received PORT. Similar to our findings, several clinicopathologic factors were found to be significant risk factors for survival in patients with head and neck ACCs. Positive resection margins were identified as an independent negative prognostic factor, especially for local control by several studies [18–22]. In addition, several studies found an association between the T stage, N stage, age, performance status, and solid histology, and dismal prognosis [23–26]. Studies that evaluated the prognostic significance of PNI have reported conflicting outcomes, with some identifying it as an important factor [5, 27, 28] and others finding no survival impact [10, 23, 25, 29, 30]. Involvement of the submandibular gland is also associated with worse survival compared to other major glands [31, 32]. Although sex has not been a very common risk factor in head and neck ACC, several studies showed that female patients have significantly better survival rates [33, 34]. These findings suggest the need to evaluate more thoroughly the possible sex differences in terms of biology and behavior of disease. Moreover, hormonal influences may have had an impact, and women could be more likely to adhere to the treatment plan or tolerate the treatment. It is also possible that women are more likely to seek medical attention and therefore received an earlier diagnosis.

Surgical resection and PORT constitute the primary tumor management for ACC. Although PORT is considered as one of the principal treatments for ACCs [8], its impact on survival remains debatable. Owing to the rarity of incidence of ACCs, randomized controlled studies to elucidate the role of PORT or determine the subset of the ACC population most likely to benefit have been limited. PORT reportedly exerts a positive impact on survival and improves local control [5, 6, 11, 21, 27, 35, 36]. Better local control is of particular importance in delaying disease progression and maintaining the quality of life [21]. Several researchers have attempted to find the subset who would derive benefits from PORT. Although the recent SEER study [32] found that PORT conferred no survival benefit in patients with early-stage and non-submandibular ACC, it was significantly beneficial in submandibular stage III ACC. Chen et al. [37] reported that the LRFFS improved significantly after PORT in intermediate and high-risk patients stratified according to T stage, margin, location, and bone invasion. However, a few studies have reported little or no benefit with PORT [9–11, 33, 34, 38].

In our study, the addition of PORT to the treatment regimen of high-risk patients, with a poor prognosis, resulted in survival comparable to that of those who did not receive PORT. Although the difference was not significant in low-risk patients, they might also derive some benefit from PORT. The discrepancy among the results of previous studies may be attributed to the variations in the study design, eligibility, follow-up duration, and sample size. We conducted a detailed analysis of the effect on the pattern of recurrences through several subgroup analyses to clarify the benefit of PORT to the greatest extent possible, and posited the potential mechanism underlying survival improvement with PORT. Moreover, we demonstrated that as local recurrences may lead to a dismal prognosis, active local therapy is required for such patients.

The radiotherapeutic approach for PORT is challenging due to the presence of numerous radiosensitive structures in the head and neck region, as doses ranging between 60 and 70 Gy should be achieved in macroscopic tumors. Recent advances in radiation oncology and treatment planning have led to the implementation of IMRT. This treatment technique allows optimization of target volume coverage, while facilitating reduction in the dose to the surrounding organs at risk. Therefore, a higher total dose could be achieved with better coverage of the target volume. Several studies using IMRT showed an improvement in local control rates and acceptable toxicities [39–41]. Due to the radioresistance of ACC and prognosis improvement after a higher RT dose (≥ 60 Gy) [21, 42], RT techniques using high linear energy transfer particles have been also evaluated. Charged particle beams such as proton therapy and carbon ion radiotherapy are characterized by a Bragg peak that enables delivery of larger doses of radiation to the tumor while significantly lowering the dose to the surrounding healthy tissues. Recently, carbon-ion radiotherapy is increasingly used for ACC irradiation, based on immunological, molecular, and clinicopathological rationale [42]. Carbon-ion radiotherapy has shown excellent local control rates in the adjuvant setting as well as in the definitive setting, without an increase in grade ≥ 3 toxicities [42–44]. Although we could not demonstrate the effectiveness of heavy-ion therapy in this study, we showed that the RT technique (IMRT) was an independent significant prognostic factor for LRFFS.

ACC is known for infrequent lymphatic spread to the neck, despite a high rate of local and distant recurrence. While therapeutic neck dissection is performed in all clinically node-positive patients, management of cN0 neck remains controversial, and elective neck dissection is not routinely performed in these patients. While isolated lymph node involvement may not have a significant effect on survival, it can be a risk factor for subsequent distant metastases [45]. According to a recent meta-analysis [46], there was no significant difference in survival between the elective neck dissection and observation arms. In addition, most of the patients do not develop regional metastases during follow-up, making elective neck dissection questionable in terms of prognosis. In our study, elective neck treatment was performed in 48% of node-negative patients, and the aim or type of neck treatment was not a significant prognostic factor. Thus, the role of elective neck treatment in initially node-negative ACC patients is still inconclusive. In addition, there is a lack of consensus on the treatment field of elective neck treatment. Nevertheless, a subgroup with a higher risk of occult neck metastasis would exist, and they would benefit from elective neck treatment. Therefore, further well-balanced studies are needed, and efforts to create an international consensus should be taken to address this issue.

Our study had several limitations. First, its retrospective design could have affected the statistical power, leading to selection bias and measurement bias. Second, a follow-up period longer than the median 84.7 months of our study may be needed. Although tumor progression usually occurs within 5 years and decreases rapidly after 5 years in other tumors, the survival rates of patients with ACC can decrease continuously for more than 10 years after initial treatment [37, 47]. Third, the prognostic importance of salvage treatment should also be addressed further. The survival period of ACC patients is usually long despite repetitive disease recurrences and limited effective salvage treatment options. In our study, the survival of patients who received local salvage treatment was significantly longer regardless of the type of recurrence. As we could not perform more detailed analyses, follow-up studies for the optimal local salvage treatment and patient selection are still needed. Therefore, more comprehensive, prospective studies with larger sample sizes are necessary in the future.

In conclusion, PORT is an important factor in improving the local control rate of head and neck ACCs. In selected patients, it is expected to prolong survival by decreasing local failures significantly. PORT should be individualized to enhance the treatment efficacy and avoid unnecessary adverse events. Larger sample sizes and longer follow-up time are required to clarify the benefit of PORT not only in low-risk, but also in high-risk patients. Our RPA model will be a useful tool for predicting the patient's prognosis in advance and selecting optimal adjuvant treatment strategies.

## Supplementary Information

**Additional file 1**. **Supplementary Table 1.** Results of univariate analyses for locoregional failure-free survival (LRFFS), progression-free survival (PFS), and overall survival (OS) in all patients. **Supplementary Table 2.** Results of multivariate analyses for locoregional failure-free survival (LRFFS), progression-free survival (PFS), and overall survival (OS) in all patients. **Supplementary Table 3.** Comparison of treatment failure rates between patients who underwent postoperative radiotherapy (PORT [+]) and those who did not undergo postoperative radiotherapy (PORT [-]). **Supplementary Figure 1.** Kaplan-Meier estimates for overall survival (a) according to whether the patient received salvage treatment or not, and (b) according to the type of salvage treatments.

## Data Availability

Research data are stored in an institutional repository and will be shared upon request to the corresponding author.
