# Supplementary material for: Role of postoperative radiotherapy in resected adenoid cystic carcinoma of the head and neck
Source: Radiat Oncol. 2022 Dec 1;17:197. doi: 10.1186/s13014-022-02165-5 (PMC9716721; doi:10.1186/s13014-022-02165-5)

Supplementary Table 1. Results of univariate analyses for locoregional failure-free survival (LRFFS), progression-free survival (PFS), and overall survival (OS) in all patients

|  | **Variables** |  | **LRFFS** | | |  | **PFS** | | |  | **OS** | | |  |
| --- | --- | --- | --- | --- | --- | --- | --- | --- | --- | --- | --- | --- | --- | --- |
|  |  |  | **HR** | **95% CI** | **P value** |  | **HR** | **95% CI** | **P value** |  | **HR** | **95% CI** | **P value** |  |
|  | Age (continuous) |  | 1.003 | 0.987-1.018 | 0.749 |  | 1.000 | 0.986-1.013 | 0.944 |  | 1.019 | 0.999-1.041 | 0.067 |  |
|  | Sex (female vs. male) |  | 0.608 | 0.381-0.970 | 0.037 |  | 0.622 | 0.416-0.931 | 0.021 |  | 0.528 | 0.297-0.940 | 0.030 |  |
|  | Location (major salivary gland vs. others) |  | 0.466 | 0.267-0.813 | 0.007 |  | 0.367 | 0.222-0.607 | <0.001 |  | 0.442 | 0.213-0.914 | 0.028 |  |
|  | Histologic grade (grade 3 vs. 1-2) |  | 2.274 | 1.211-4.269 | 0.011 |  | 2.210 | 1.265-3.869 | 0.006 |  | 3.217 | 1.613-6.413 | 0.009 |  |
|  | Tumor size (continuous) |  | 1.288 | 1.127-1.469 | <0.001 |  | 1.283 | 1.146-1.437 | <0.001 |  | 1.355 | 1.168-1.574 | <0.001 |  |
|  | Tumor size (>2.5cm vs. others) |  | 2.079 | 1.286-3.361 | 0.003 |  | 2.307 | 1.522-3.497 | <0.001 |  | 2.522 | 1.376-4.622 | 0.003 |  |
|  | T stage |  | 1.344 | 1.155-1.565 | <0.001 |  | 1.370 | 1.209-1.553 | <0.001 |  | 1.445 | 1.223-1.708 | <0.001 |  |
|  | T2-4 vs. T1 |  | 3.936 | 1.876-8.259 | <0.001 |  | 3.782 | 2.061-6.938 | <0.001 |  | 25.339 | 3.463-185.4 | 0.001 |  |
|  | T3-4 vs. T1-2 |  | 2.181 | 1.366-3.483 | 0.001 |  | 2.745 | 1.827-4.123 | <0.001 |  | 3.176 | 1.764-5.718 | <0.001 |  |
|  | T4 vs. T1-3 |  | 2.588 | 1.587-4.220 | <0.001 |  | 2.712 | 1.766-4.162 | <0.001 |  | 3.140 | 1.750-5.635 | <0.001 |  |
|  | N stage |  | 1.610 | 1.046-2.479 | 0.031 |  | 1.583 | 1.041-2.407 | 0.032 |  | 2.297 | 1.482-3.562 | <0.001 |  |
|  | N1-2 vs. N0 |  | 1.612 | 1.047-2.482 | 0.030 |  | 1.444 | 0.699-2.980 | 0.321 |  | 2.672 | 1.190-5.996 | 0.017 |  |
|  | ECE |  | 2.085 | 0.954-4.556 | 0.065 |  | 2.345 | 1.134-4.847 | 0.021 |  | 3.741 | 1.666-8.399 | 0.001 |  |
|  | Surgical margin |  | 0.559 | 0.379-0.824 | 0.003 |  | 0.514 | 0.367-0.721 | <0.001 |  | 0.510 | 0.309-0.840 | 0.008 |  |
|  | Positive margin (yes vs. no) |  | 2.638 | 1.559-4.465 | <0.001 |  | 2.576 | 1.648-4.029 | <0.001 |  | 3.131 | 1.556-6.298 | 0.001 |  |
|  | PNI |  | 1.243 | 0.778-1.984 | 0.362 |  | 1.378 | 0.920-2.065 | 0.120 |  | 1.629 | 0.910-2.971 | 0.100 |  |
|  | LVI |  | 0.196 | 0.780-1.984 | 0.523 |  | 2.001 | 1.240-3.228 | 0.005 |  | 1.750 | 0.889-3.443 | 0.105 |  |
|  | Nerve invasion |  | 1.960 | 0.937-4.100 | 0.074 |  | 2.048 | 1.089-3.851 | 0.026 |  | 1.593 | 0.627-4.043 | 0.328 |  |
|  | Bone invasion |  | 3.170 | 1.970-5.100 | <0.001 |  | 3.559 | 2.343-5.407 | <0.001 |  | 3.858 | 2.167-6.866 | <0.001 |  |
|  | Postop RT |  | 1.017 | 0.622-1.663 | 0.947 |  | 1.687 | 1.068-2.665 | 0.025 |  | 3.315 | 1.484-7.404 | 0.004 |  |
|  | Neck treatment (yes vs. no) |  | 0.841 | 0.527-1.341 | 0.466 |  | 1.085 | 0.722-1.630 | 0.696 |  | 1.030 | 0.579-1.833 | 0.920 |  |
|  | Aim of neck treatment |  | 0.884 | 0.694-1.125 | 0.317 |  | 1.017 | 0.826-1.252 | 0.876 |  | 0.925 | 0.688-1.244 | 0.607 |  |
|  | Type of neck treatment |  | 0.867 | 0.715-1.050 | 0.144 |  | 0.922 | 0.793-1.074 | 0.297 |  | 1.040 | 0.843-1.282 | 0.714 |  |

***Abbreviations:*** LRFFS, locoregional failure-free survival; PFS, progression-free survival; OS, overall survival; HR, hazard ratio; CI, confidence interval; ECE, extracapsular extension; PNI, perineural invasion; LVI, lymphovascular invasion; RT, radiotherapy

Supplementary Table 2. Results of multivariate analyses for locoregional failure-free survival (LRFFS), progression-free survival (PFS), and overall survival (OS) in all patients

|  | **Endpoint** | **Variables** |  | **HR** | **95% CI** | | **P value** |  |
| --- | --- | --- | --- | --- | --- | --- | --- | --- |
|  | **LRFFS** | Sex (female vs. male) |  | 0.603 | 0.366 | 0.994 | 0.047 |  |
|  |  | Major salivary gland |  | 0.519 | 0.279 | 0.965 | 0.038 |  |
|  |  | Histologic grade (3 vs. 1-2) |  | 1.815 | 0.937 | 3.516 | 0.077 |  |
|  |  | T2-4 vs. T1 |  | 0.341 | 0.142 | 0.816 | 0.016 |  |
|  |  | N1-2 vs. N0 |  | 1.587 | 0.655 | 3.845 | 0.306 |  |
|  |  | Positive margin (yes vs. no) |  | 2.199 | 1.247 | 3.879 | 0.006 |  |
|  |  | Tumor size (>2.5 cm vs. others) |  | 1.200 | 0.679 | 2.120 | 0.530 |  |
|  | **PFS** | Sex (female vs. male) |  | 0.528 | 0.339 | 0.821 | 0.005 |  |
|  |  | Major salivary gland |  | 0.355 | 0.199 | 0.633 | <0.001 |  |
|  |  | Histological grade (3 vs. 1-2) |  | 1.551 | 0.851 | 2.826 | 0.152 |  |
|  |  | T2-4 vs. T1 |  | 0.339 | 0.161 | 0.716 | 0.005 |  |
|  |  | ECE |  | 2.713 | 1.091 | 6.747 | 0.032 |  |
|  |  | Positive margin (yes vs. no) |  | 1.864 | 1.159 | 2.999 | 0.010 |  |
|  |  | LVI |  | 1.514 | 0.883 | 2.595 | 0.132 |  |
|  |  | Tumor size (>2.5 cm vs. others) |  | 1.371 | 0.836 | 2.250 | 0.211 |  |
|  | **OS** | Sex (female vs. male) |  | 0.512 | 0.272 | 0.963 | 0.038 |  |
|  |  | Major salivary gland |  | 0.480 | 0.199 | 1.157 | 0.102 |  |
|  |  | Histological grade (3 vs. 1-2) |  | 2.177 | 1.046 | 4.532 | 0.038 |  |
|  |  | T2-4 vs. T1 |  | 17.681 | 2.273 | 137.500 | 0.006 |  |
|  |  | ECE |  | 4.452 | 1.575 | 12.585 | 0.005 |  |
|  |  | Positive margin (yes vs. no) |  | 2.327 | 1.077 | 5.028 | 0.032 |  |
|  |  | Tumor size (>2.5 cm vs. others) |  | 0.987 | 0.498 | 1.953 | 0.969 |  |

***Abbreviations:*** LRFFS, locoregional failure-free survival; PFS, progression-free survival; OS, overall survival; HR, hazard ratio; CI, confidence interval; ECE, extracapsular extension; LVI, lymphovascular invasion

Supplementary Table 3. Comparison of treatment failure rates between patients who underwent postoperative radiotherapy (PORT [+]) and those who did not undergo postoperative radiotherapy (PORT [-])

|  |  |  | **PORT (+)** | | **PORT (-)** | |  |  |
| --- | --- | --- | --- | --- | --- | --- | --- | --- |
|  |  |  | **No.** | **%** | **No.** | **%** | **P value** |  |
|  | Pattern of first recurrence |  |  |  |  |  |  |  |
|  | Local |  | 11 | 8.9 | 13 | 20.6 | 0.001 |  |
|  | Regional |  | 3 | 2.4 | 1 | 1.6 |  |  |
|  | Distant |  | 44 | 35.5 | 5 | 7.9 |  |  |
|  | Local + distant |  | 4 | 3.2 | 4 | 6.3 |  |  |
|  | Regional + distant |  | 0 | 0.0 | 1 | 1.6 |  |  |
|  | Any recurrence |  | 64 | 51.6 | 24 | 38.1 | 0.080 |  |
|  | Local failure |  | 21 | 16.9 | 20 | 31.7 | 0.021 |  |
|  | Regional failure |  | 5 | 4.0 | 2 | 3.2 | 0.770 |  |
|  | Locoregional failure |  | 22 | 17.7 | 21 | 33.3 | 0.017 |  |
|  | Distant failure |  | 52 | 41.9 | 15 | 23.8 | 0.015 |  |

***Abbreviations:*** PORT, postoperative radiotherapy

Supplementary Figure 1. Kaplan-Meier estimates for overall survival (a) according to whether the patient received salvage treatment or not, and (b) according to the type of salvage treatments


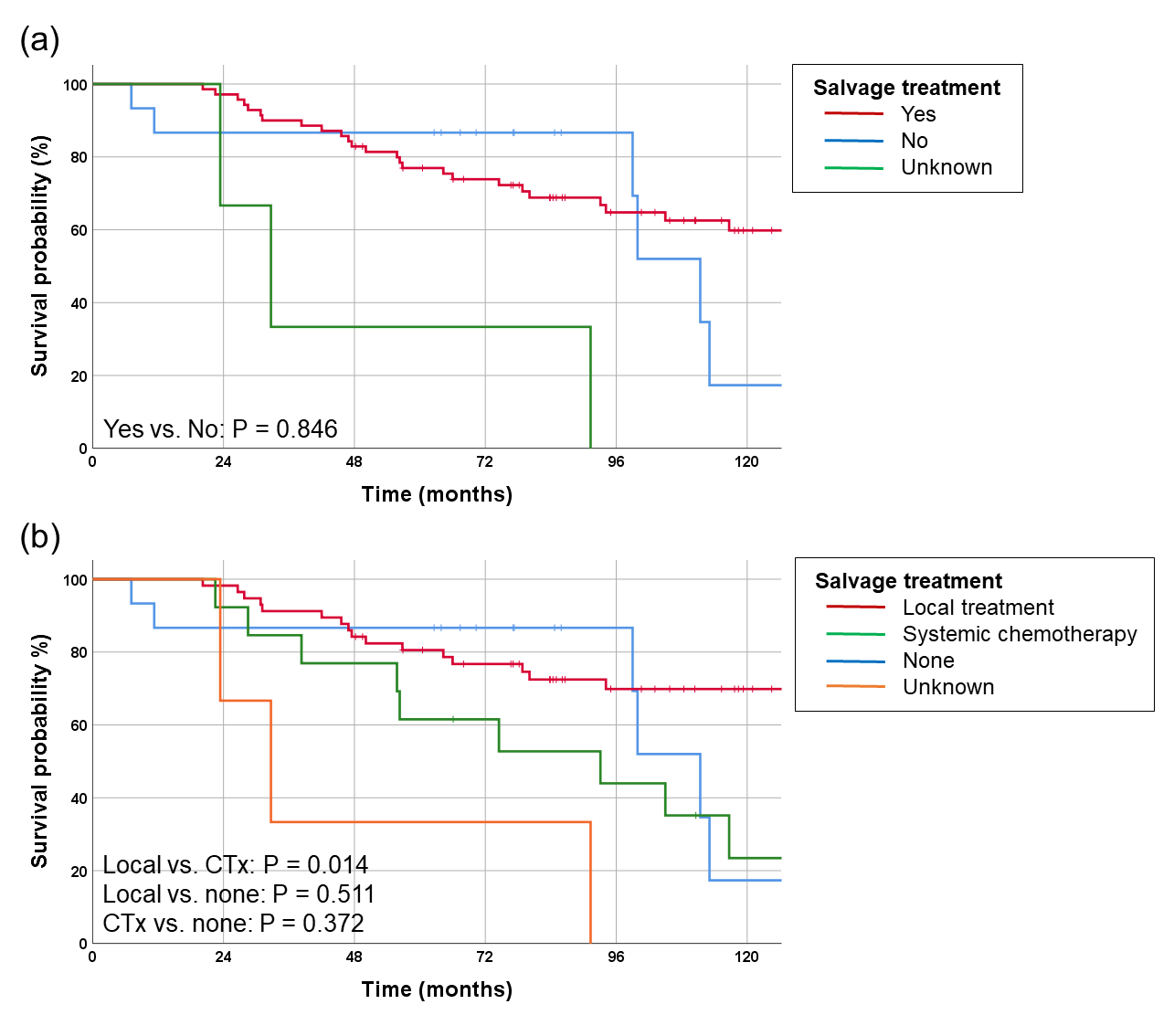

Supplement: Supplementary file 1 — Additional file 1. Supplementary Table 1. Results of univariate analyses for locoregional failure-free survival (LRFFS), progression-free survival (PFS), and overall survival (OS) in all patients. Supplementary Table 2. Results of multivariate analyses for locoregional failure-free survival (LRFFS), progression-free survival (PFS), and overall survival (OS) in all patients. Supplementary Table 3. Comparison of treatment failure rates between patients who underwent postoperative radiotherapy (PORT [+]) and those who did not undergo postoperative radiotherapy (PORT [-]). Supplementary Figure 1. Kaplan-Meier estimates for overall survival (a) according to whether the patient received salvage treatment or not, and (b) according to the type of salvage treatments. [file 13014_2022_2165_MOESM1_ESM.docx]
